# The Role of Interferon Antagonist, Non-Structural Proteins in the Pathogenesis and Emergence of Arboviruses

**DOI:** 10.3390/v3060629

**Published:** 2011-06-01

**Authors:** Bradley S. Hollidge, Susan R. Weiss, Samantha S. Soldan

**Affiliations:** 1 Department of Neurology, University of Pennsylvania School of Medicine, Philadelphia, PA 19104, USA; E-Mail: hollidge@mail.med.upenn.edu; 2 Neuroscience Graduate Group, University of Pennsylvania School of Medicine, Philadelphia, PA 19104, USA; 3 Department of Microbiology, University of Pennsylvania School of Medicine, Philadelphia, PA 19104, USA; E-Mail: weisssr@mail.med.upenn.edu

**Keywords:** arbovirus, interferon, emergence, innate immune system, bunyavirus, flavivirus, alphavirus

## Abstract

A myriad of factors favor the emergence and re-emergence of arthropod-borne viruses (arboviruses), including migration, climate change, intensified livestock production, an increasing volume of international trade and transportation, and changes to ecosystems (e.g., deforestation and loss of biodiversity). Consequently, arboviruses are distributed worldwide and represent over 30% of all emerging infectious diseases identified in the past decade. Although some arboviral infections go undetected or are associated with mild, flu-like symptoms, many are important human and veterinary pathogens causing serious illnesses such as arthritis, gastroenteritis, encephalitis and hemorrhagic fever and devastating economic loss as a consequence of lost productivity and high mortality rates among livestock. One of the most consistent molecular features of emerging arboviruses, in addition to their near exclusive use of RNA genomes, is the inclusion of viral, non-structural proteins that act as interferon antagonists. In this review, we describe these interferon antagonists and common strategies that arboviruses use to counter the host innate immune response. In addition, we discuss the complex interplay between host factors and viral determinants that are associated with virus emergence and re-emergence, and identify potential targets for vaccine and anti-viral therapies.

## 1. Introduction

Arthropod-borne viruses (arboviruses) are a large group of viruses that use hematophagous (blood feeding) arthropod vectors, including mosquitoes, ticks, biting midges, and phlebotomine flies, for transmission between vertebrate hosts. Arboviruses come from numerous viral families, including *Togaviridae* (genus *Alphavirus*); *Bunyaviridae* (genera *Nairovirus*, *Orthobunyavirus*, and *Phlebovirus*); *Flaviviridae* (genus *Flavivirus*); *Rhabdoviridae* (genus *Vesiculovirus*); *Orthomyxoviridae* (genus *Thogotovirus*); *Reoviridae* (genus *Orbivirus*); and *Asfarviridae* (genus *Asfarvirus*) [1,2]. In addition, a diverse group of arthropod-vectored plant viruses have been described including members of the *Bunyaviridae* (genus *Tospovirus*); *Geminiviridae* (genera *Mastrevirus*, *Curtovirus*, *Begomovirus*, and *Topocuvirus*); *Caulimoviridae* (genera *Badnavirus*, *Caulimovirus*, *Tungrovirus*, and *Soymovirus*); *Potyviridae* (genera *Potyvirus*, *Rymovirus*, *Macluravirus*, *Ipomovirus*, and *Tritimovirus*); and *Closteroviridae* (genera *Closterovirus*, *Crinivirus*, and *Ampelovirus*). However, because they do not infect vertebrates, these arthropod-vectored plant viruses are not classified as arboviruses [3–5]. Remarkably, arboviruses have the ability to infect a broad range of vertebrate hosts, which is important for their maintenance and amplification in nature; these amplifying vertebrate hosts achieve a high viremia that enables transmission of the virus to a naïve arthropod taking a blood meal. Despite this high viremia, most amplifying hosts do not typically show overt signs of disease because of these infections.

In most instances, humans serve as dead-end hosts and do not participate in the maintenance of arbovirus lifecycles. Unlike amplifying hosts, humans and other dead-end hosts can develop disease caused by arboviral infection. Although many arboviral infections in humans are asymptomatic or result in a mild influenza-like illness, some arboviruses can cause respiratory illness, arthritis, febrile illness, encephalitis, hemorrhagic syndrome, shock, and/or death. Therefore, several arboviruses have become increasingly important human and veterinary pathogens, representing nearly 30% of all emerging infectious diseases in the last decade [6]. In this review, part of a special issue on the pathogenesis of emerging and re-emerging RNA viruses, we will focus on members of the genera *Flavivirus* and *Alphavirus* and the family *Bunyaviridae* and the role of their nonstructural proteins in antagonizing the host interferon (IFN) response.

## 2. Emergence

Arthropod vectors are both direct and indirect factors in the emergence and re-emergence of many arboviruses. The sylvatic transmission cycle between the invertebrate vector(s) and vertebrate reservoir not only allows for the maintenance and amplification of the arboviruses, but also contributes to their emergence. In addition, climate plays a critical role in determining the transmission patterns for arboviruses. The virus can either circulate throughout most of the year with broad seasonal peaks in tropical areas or, in a more temperate climate, it can be transmitted between vectors and vertebrates during the warmer months while overwintering in mosquito eggs [7]. In these temperate climates, arboviral disease is absent during the colder months. Longer periods of warm weather not only lengthen the seasonal peaks of virus circulation, but also provide conditions conducive to increasing vector populations. Climate change is thought to exacerbate the emergence and re-emergence of arboviruses dependent on these periods of warm weather and provide opportunities for changes in vector range, vertebrate host and vector composition [1,8].

Human behavior influences arthropod vectors in a variety of ways that contribute to the emergence and re-emergence of arboviruses. Urban sprawl, population growth, and agricultural development have increased human contact with arboviral vectors. Population growth and urbanization have led to crowded living conditions and provided ideal breeding sites for mosquitoes owing to the inadequate management of water and waste, further increasing the intersection of dense human and vector populations. Globalization, including modern travel and trade, has facilitated the spread of arboviruses and the anthropophilic mosquitoes, including *Aedes aegypti*, *Aedes albopictus*, and mosquitoes in the *Culex pipiens* complex, which have the potential to introduce arboviruses into a naïve population of vertebrate hosts [9,10]. Although the spread of arboviruses linked to travel has not been directly attributed to infected humans seeding the virus in naïve populations, this possibility/scenario is of growing concern because some arboviruses have been able to adapt to an urban epidemic cycle in which humans have become the primary amplifying host; an urban epidemic cycle has been described for dengue virus (DENV), yellow fever virus (YFV), and chikungunya virus (CHIKV) [11–14]. The worldwide distribution and potential for emergence of arboviruses along with the paucity of effective vaccines and therapeutics underscores the importance of these viruses in the increasingly globalized human population.

## 3. Interferon Responses to Viral Infection

With the exception of the *Asfarviridae*, a family of DNA viruses that replicate in ticks (its role in countering the innate immune system is reviewed in [15]), arboviruses are single stranded RNA viruses. Type I IFNs are induced by dsRNA as well as viral RNA containing 5′-triphosphates and it has been demonstrated that many arboviruses induce an IFN response shortly following delivery from their arthropod vector. The initial interaction between viruses and the innate immune system plays a major role in determining whether the virus will successfully establish infection. To counter the host immune response, many arboviruses encode nonstructural as well as some structural proteins like the capsid of the equine encephalitis viruses, that function, in part, to antagonize the host IFN response [16,17]. The focus of this review will solely be on the nonstructural proteins of arboviruses that serve as IFN antagonists (Table 1). This adaptation has enhanced the ability of these viruses to establish themselves in a multitude of vertebrate hosts and plays an important role in their emergence. Importantly, a greater understanding of how these arboviral nonstructural proteins counter the host immune response may lead to the development of attenuated vaccines and/or antiviral therapies. Accordingly, arboviral nonstructural proteins that counteract IFNs are the focus of this review.

**Figure 1.**
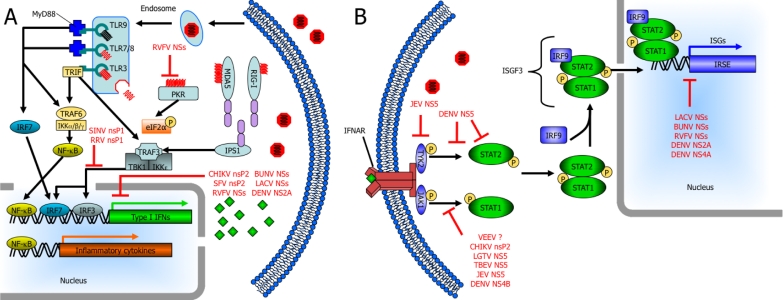
Arbovirus nonstructural proteins use several mechanisms to disrupt IFN induction and signaling. (**A**) The Toll-like receptor (TLR) family members, TLR3, TLR7, TLR8, and TLR9 are located in endosomal membranes sensing double-stranded RNA (dsRNA), single-stranded RNA (ssRNA), and CpG DNA ligands, respectively, in the endosomes. TLR7, TLR8, and TLR9 signal through the MyD88 adapter ultimately leading to production of type I IFNs and inflammatory cytokines. TLR3 uses the adapter protein Toll/interleukin-1 (IL-1) receptor (TIR) domain-containing adapter-inducing IFN-β (TRIF) inducing production of type I IFNs. Retinoic acid-inducible gene I (RIG-I), melanoma differentiation-associated gene 5 (MDA5) and double-stranded RNA-dependent protein kinase (PKR) detect viral products in the cytosol. Both MDA5 and RIG-I signal through the mitochondrial-membrane-associated IFN-β promoter stimulator 1 (IPS1) resulting in activation of IRF3 and IRF7. (**B**) Secreted IFN-α/β binds in an autocrine or paracrine fashion to cell surface IFN-α receptors (IFNAR). Subsequently, JAK-STAT signaling leads to phosphorylation and dimerization of signal transducer and activator of transcription 1 (STAT1) and STAT2, the formation of the IFN-stimulated gene factor 3 (ISGF3) complex and its translocation to the nucleus. Here, ISGF3 binds to IFN-stimulated response elements (ISREs) and initiates transcription of IFN-stimulated genes (ISGs). Bunyavirus NSs (nonstructural protein encoded by the S segment) targets the activities of RNA polymerase II and many other arboviruses inhibit JAK-STAT signaling. Nonstructural proteins can cause the degradation of some of the components involved in these pathways. Although not the focus of this review, other arboviral proteins, including New World alphavirus capsid proteins, can also play a role in IFN antagonism.

The rapid production of IFNs and other cytokines are important components of the innate immune response against viral infections and ultimately, the clearance of the infection. In the cell, IFN induction due to cytoplasmic signaling cascades triggered by the detection of viral components and the resulting IFN-α/β induces an antiviral state alleviating virus burden and initiating an adaptive immune response. For RNA viruses to replicate, viral genomic RNA must be unwrapped from the nucleocapsid and exposed to allow for viral transcription/translation. However, exposed viral RNA provides a reliable substrate for cells to sense a viral infection. Both retinoic acid-inducible gene I (RIG-I) and melanoma differentiation-associated gene 5 (MDA5) are cytoplasmic proteins that recognize viral components in the cytosol. RIG-I preferentially binds 5′-phosphorylated ssRNA and short dsRNA while MDA5 does not require 5′-phosphorylation and is activated by higher-order structured RNA containing ssRNA or dsRNA [18–25]. MDA5 and RIG-I signal through mitochondrial membrane-associated INF-β promoter stimulator 1 (IPS-1). Subsequently, tumor necrosis factor (TNF) receptor-associated factor 3 (TRAF3) activates TANK-binding kinase 1 (TBK1) and inhibitor of nuclear factor kappa B (NF-κB) kinase ɛ (IKKɛ). TBK1 and IKKɛ phosphorylate IFN regulatory factor 3 (IRF-3) and IRF-7, which translocate into the nucleus where they bind IFN-stimulated response elements (ISREs) resulting in type I IFN expression, namely IFN-β and IFN-α4 as early type I IFNs (Figure 1) [26–28].

Additionally, mammalian cells use transmembrane proteins known as toll-like receptors (TLRs) to sense pathogen-associated molecular patterns. There are 10 members of the TLR family in humans and each one is capable of detecting a specific pathogen-associated molecular pattern [29–31]. TLR3, TLR7, TLR8, and TLR9 are the main viral sensors and they are located on the cell surface (TLR3) or in endosomal compartments (TLR3, TLR7, TLR8, and TLR9). TLR7 and TLR8 detect ssRNA. The specificity of the dsRNA receptor TLR3 allows it to recognize genomes of dsRNA viruses as well as replicative intermediates of ssRNA viruses [32–36]. Unmethylated CpG-containing DNA, a common component in the genomes of DNA viruses, is recognized by TLR9 [37]. TLR7, TLR8, and TLR9 signal through the MyD88 adapter leading to activation of type I IFNs and NF-κB signaling, while TLR3 signals through the adapter protein TIR domain-containing adapter-inducing IFN-β (TRIF) which results in type I IFN induction (Figure 1) [38].

Two other cellular sensors are protein kinase R (PKR) and 2′,5′-oligoadenylate synthetase (OAS). PKR dimerizes upon binding dsRNA and subsequently phosphorylates eukaryotic initiation factor 2-α (eIF2-α) preventing viral replication by inhibition of translation and activates NF-κB by degradation of IκB [39]. 2′,5′-OAS is activated by dsRNA initiation ultimately leading to activation of RNase L, an endoribonuclease, to degrade RNA [40]. Importantly, many of these viral sensors are up-regulated in response to type I IFNs.

Early type I IFNs bind to cell surface IFN-α/β receptors (IFNARs), which are heterodimers composed of two subunits, IFNAR1 and IFNAR2 (Figure 1) [41]. The janus kinases Tyk2 and Jak1 are associated with the cytoplasmic tails of IFNARs and are activated by IFN binding to its receptor [42–44]. Subsequently, STAT2 is recruited by phosphorylated tyrosine residues on the IFNAR and becomes phosphorylated (pSTAT2) [45]. Active pSTAT2 recruits and phosphorylates STAT1 (pSTAT1) [46]. pSTAT1 and pSTAT2 heterodimerize and complex with IRF-9, forming the transcription factor complex IFN-stimulated gene factor 3 (ISGF3), which translocates to the nucleus activating ISREs [47–49]. These ISREs are within upstream promoter regions of IFN-stimulated genes (ISGs) and regulate their expression. ISGs include viral pattern recognitions receptors such as RIG-I, MDA5, TLRs, 2′,5′-OAS, PKR; transcription factors such as STAT1, STAT2, IRF3 as well as antiviral effectors such as Mx and Ifit family members that are important for the control of viral infection. Thus, viruses use numerous strategies to antagonize the IFN response since expression of ISGs is critical in limiting viral replication. These strategies used by arboviruses will be discussed in the context of bunyavirus, alphavirus, and flavivirus nonstructural proteins.

## 4. Bunyaviruses

Five genera (*Orthobunyavirus*, *Hantavirus*, *Nairovirus*, *Phlebovirus*, and *Tospovirus*) make up one of the largest and most diverse viral families, the *Bunyaviridae* [50]. Bunyaviruses are disseminated worldwide and infect a broad range of invertebrate and vertebrate hosts. With the exception of the hantaviruses [51], all of the genera within the *Bunyaviridae* are vector-borne [50]. While the tospoviruses are plant pathogens, the majority of bunyaviruses are significant pathogens in humans and animals. Notably, Crimean-Congo hemorrhagic fever virus (CCHFV; *Nairovirus*), La Crosse virus (LACV; *Orthobunyavirus*), and Rift Valley fever virus (RVFV; *Phlebovirus*) are important human pathogens causing disease ranging from mild, febrile illness to more severe disease including pulmonary disease, hemorrhagic fever, and fatal encephalitis [52,53]. The potential for emergence of these viruses is demonstrated by CCHFV, which is the second most widespread of all medically important arboviruses after DENV and is endemic in much of Africa, Asia, and Europe [54,55]. With the exception of vaccines for veterinary use to protect against RVFV, there are currently no effective therapeutics or vaccines for bunyaviruses.

**Figure 2.**
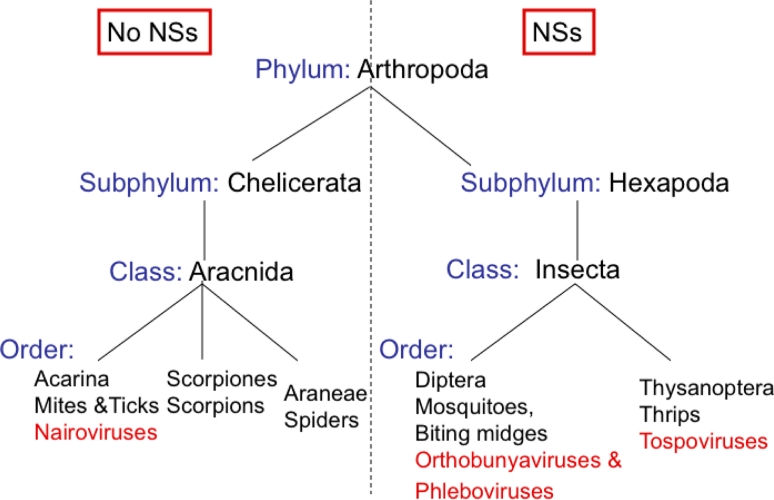
Phylogeny of arthropod vectors used by the *Bunyaviridae*. Bunyaviruses whose life cycle includes an insect vector (phleboviruses, orthobunyaviruses, and tospoviruses) encode a NSs protein either through an ambisense coding mechanism or in a reading frame that overlaps with that of the nucleocapsid (N). In contrast, bunyaviruses that do not use insect vectors (nairoviruses and hantaviruses) lack a NSs protein.

The bunyavirus genome consists of three single-stranded, negative-sense RNA segments designated by size as large (L), medium (M), and small (S). The L segment encodes the viral polymerase. A polyprotein, encoded in a single open reading frame by the M segment, is post-translationally processed into the viral glycoproteins, Gc and Gn, and in the case of the orthobunyaviruses and some phleboviruses, a nonstructural protein (NSm) of unknown function. The S segment encodes the nucleocapsid protein (N). For three bunyavirus genera, the S segment also encodes another nonstructural protein (NSs) through an overlapping reading frame in most, but not all, orthobunyaviruses or an ambisense coding mechanism (phleboviruses and tospoviruses) [56,57]. Interestingly, there is a clear divergence among the bunyaviruses delineated by the presence or absence of NSs. Bunyaviruses that use insects as their vectors (orthobunyaviruses, phleboviruses, and tospoviruses) encode NSs proteins while those bunyaviruses that use non-insect vectors (nairoviruses) or are not vector-borne (hantaviruses) do not encode NSs proteins (Figure 2). The bunyavirus maintenance and amplification cycles, alternating between insect vectors and vertebrate hosts, demonstrates this group’s ability to adapt and take advantage of the host immune system. For example, while LACV infection in insect cells is noncytolytic and leads to long-term viral persistence, it causes host cell gene expression shutoff at the level of transcription and cell death in mammalian cells resulting in cell lysis or cell death [58,59]. Mosquitoes, as well as other insects, have an effective innate immune system comprised of three major signaling pathways, the Toll, the Immune Deficiency (imd), and the JAK-STAT pathways [60–64]. When LACV-directed siRNAs against the L, M, or S segments were transfected into mammalian 293T and insect C6/36 cells, the siRNA targeting the LACV S segment was the most effective inhibitor of virus replication in both cell types [65]. These data suggest a conserved function of NSs in both mammalian and insect cells. We speculate that if the NSs inhibits the JAK-STAT pathway in insects, then the virus may be able to sustain an infection. Furthermore, the absence of cytolytic infection in insect cells and the lack of adaptive immune responses in insects may allow the virus to persist in its insect hosts. However, bunyaviruses lacking NSs protein avoid RIG-I activation by removing the 5′-triphosphates from their genomes to avoid IFN induction [66]. Therefore, viruses lacking a NSs may not have been subject to the same selective pressure to express a potent IFN antagonist.

### 4.1. Orthobunyaviridae: La Crosse Virus (LACV) and Bunyamwera Virus (BUNV)

Within the *Orthobunyavirus* genus, the California serogroup consists of 14 viruses that are antigenically related to its type species, California encephalitis virus. Some members of the California serogroup can lead to neuronal infection and encephalitis following peripheral infection in mice and humans [50,67]. LACV is the most studied of the California serogroup and is an emerging cause of pediatric encephalitis and aseptic meningitis in the American South [68,69]. While cases of LACV encephalitis have historically occurred in the midwestern United States, LACV activity has risen above endemic levels in the southeastern United States including West Virginia, North Carolina, and Tennessee [68]. The isolation of LACV from *Ae. albopictus* mosquitoes in Texas has led to growing concern since the virus has adapted from this invasive mosquito species [70].

The LACV NSs suppresses the type I IFN system in mammalian cells [71] and the use of a recombinant LACV mutant lacking NSs (rLACVΔNSs) has provided an important tool in determining the role of NSs in LACV pathogenesis. Infecting insect cells with rLACVΔNSs, Blakqori *et al.* [71] demonstrated that NSs is not required for infection or virus growth, and does not inhibit RNAi. However, in IFN-competent mouse embryonic fibroblasts, rLACVΔNSs strongly induces transcription of the IFN-β gene while wild-type recombinant LACV (WT rLACV) does not as measured by RT-PCR. IFN induction by LACV mainly occurs through the signaling pathway initiated by RIG-I [72]. Furthermore, mouse embryonic fibroblasts lacking the IFNAR (IFNAR −/−) still produced IFN-β following infection with rLACVΔNSs, demonstrating that IFN induction is a direct result of virus infection and not due to secreted IFN [71]. While there was no quantitation of the RT-PCR provided, the LACV N gene showed that viruses replicated to similar levels, cellular β-actin mRNA indicated similar amounts of RNA in the preparations and it appears the IFN-β gene was equally activated in both WT and IFNAR−/− mouse embryonic fibroblasts when infected with rLACVΔNSs. The authors confirmed that the block in IFN induction is solely due to NSs by transfecting human 293 cells with a NSs-expressing plasmid and an IFN-β promoter reporter plasmid. The cells were stimulated with synthetic dsRNA and LACV NSs potently inhibited the induction of the IFN-β promoter in response to dsRNA. Additionally, in both cell culture and mice, wt rLACV had a growth advantage over rLACVΔNSs that was abrogated in IFNAR −/− mouse embryonic fibroblasts and mice [71]. Therefore, NSs is only important in IFN-competent mammalian hosts and the lack of the LACV NSs gene can be compensated for by the inactivation of the host’s IFN system. Importantly, brains of mice infected with rLACVΔNSs induced significantly higher IFN-β and IFN-α transcripts than WT rLACV demonstrating that IFN antagonism by NSs of LACV is also functional in neurons and may play a critical role in the neuropathogenesis of LACV infection [71]. Importantly, both viruses (WT rLACV and rLACVΔNSs) preferentially infected neurons and no viral antigen was detected in GFAP positive astrocytes, oligodendrocytes, or macrophages [71] suggesting the neurons are responsible for the production of IFN-β and IFN-α in the central nervous system (CNS) of LACV infected mice.

The growth advantage of WT rLACV over rLACVΔNSs in immunocompetent mouse embryonic fibroblasts and mice [71] may demonstrate the selective pressure on LACV by the host IFN system to develop measures to counteract the IFN response. The evolution of LACV NSs IFN suppression not only provides a growth advantage to LACV, but it contributes to the pathogenesis of LACV as well. By disabling the IFN response, LACV can replicate more efficiently in cells and prevent the antiviral state of other cells, providing additional rounds of viral replication. Ultimately, this strategy allows LACV to grow to a higher titer in the peripheral tissue resulting in its neuroinvasive phenotype. Therefore, if LACV were to evolve an additional mechanism of IFN antagonism to work synergistically with the current mechanism of LACV NSs the result could lead to higher risk of neurological disease by allowing more efficient peripheral replication and/or more severe neurological disease once LACV has reached the CNS.

In the mouse model of LACV encephalitis, a high percentage of WT mice succumbed to LACV infection even when rLACVΔNSs was used [71], demonstrating that deletion of NSs was not sufficient to completely attenuate LACV *in vivo*. The authors speculated that this is because the mice lacked a suitable IFN-induced antiviral effector activity. More specifically, human MxA protein is the most effective IFN-induced effector protein against LACV and has the potential to inhibit a wide range of RNA viruses in cell culture [73,74], but the murine homologue of human MxA, Mx2, is defective in most inbred mouse strains including the C57BL/6 mice used in their experiments [71,75]. MxA-transgenic IFNAR−/− mice demonstrate an increased survival relative to IFNAR−/− mice infected with LACV. However, only about 40% of the MxA-transgenic IFNAR−/− mice survived [74], which could be due to low levels of MxA expression in muscle cells and the non-uniform expression of MxA in the brain of the MxA-transgenic lines [74,76]. Thus, while MxA is the most effective IFN-induced effector protein against LACV, it is not solely responsible for anti-LACV activity. Even in the absence of Mx genes, the IFN response still delays bunyavirus replication [77–80] and PKR has been shown to be effective against BUNV [81] and RVFV lacking NSs (see below) [78]. Since most LACV infections in humans are subclinical with only a small proportion of infected children developing encephalitis, the clinical outcome of LACV infection could be dependent on the degree of IFN production and the magnitude of IFN-induced antiviral effector proteins, including but not limited to MxA or PKR, in limiting peripheral LACV replication and virus spread to the CNS.

**Figure 3.**
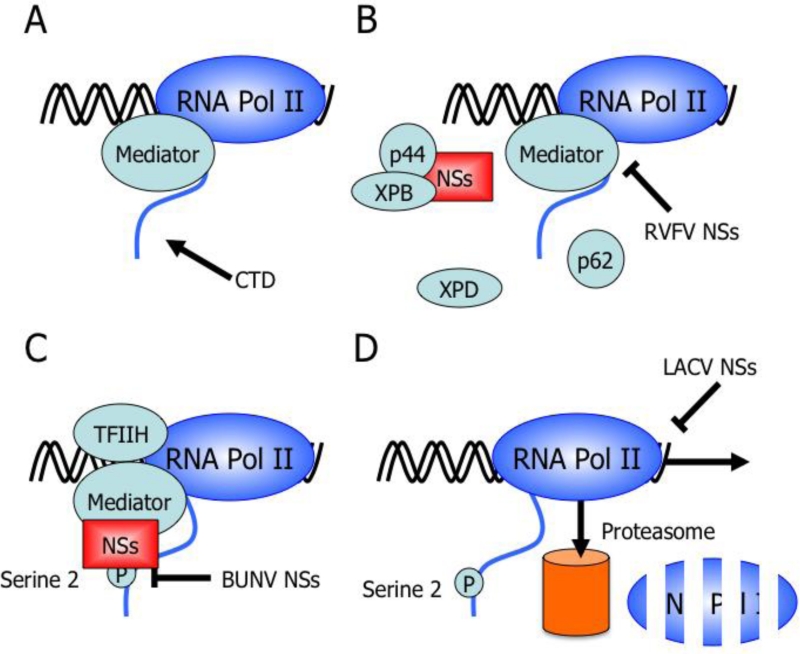
Mechanisms of bunyavirus NSs proteins targeting RNA polymerase II transcription to counteract interferon gene expression. (**A**) The Mediator complex binds to the C-terminal domain (CTD) of RNA polymerase II holoenzyme. Transcription factors and RNA polymerase associate with Mediator and allow for the formation of the preinitiation complex. (**B**) Rift Valley fever virus (RVFV) NSs binds p44, a subunit of TFIIH, preventing the assembly of newly synthesized TFIIH or recycled TFIIH subcomplexes [83]. RVFV NSs forms nuclear filamentous structures that also contain TFIIH subunits p44 and XPB [83]. Therefore, RVFV NSs prevents the assembly of the transcription preinitiation complex. (**C**) Bunyamwera virus (BUNV) NSs prevents the phosphorylation of serine 2 of the CTD of RNA polymerase II. Phosphorylation of serine 2 is for mRNA elongation and 3′-end processing suggesting BUNV NSs inhibits RNA polymerase II transcription by preventing the transition from initiation to elongation [84,85]. (**D**) La Crosse virus (LACV) NSs induces a DNA damage-like response triggering the degradation of RPB1 [72]. This mechanism specifically targets RPB1 from transcribing RNA polymerase and thus requires the phosphorylation of serine 2 on the CTD of RNA polymerase II [72].

RT-PCR analysis for some RNA polymerase II-dependent cellular genes that are constitutively expressed demonstrated a strong reduction in WT LACV infected cells, suggesting that LACV NSs may also affect host cell transcription similar to bunyamwera virus (BUNV) and RVFV [71,82–85]. Causing generalized inhibition of host cell transcription and thus gene expression is an effective way for viruses to block the production of IFNs and the up-regulation of IFN-induced antiviral effector proteins allowing the virus to overcome the host innate immune response. Most recently, LACV NSs was shown to act downstream of IRF-3 and specifically block RNA polymerase II transcription [72]. LACV NSs achieves this transcriptional block by inducing the degradation of RPB1, a subunit of RNA polymerase II [72]. Interestingly, transcriptional inhibition by LACV NSs is similar to the cellular DNA damage response. Both up-regulation of the DNA damage response gene pak6 and phosphorylation of histone H2A.X, one of the first events in the DNA damage response, were induced by LACV NSs [72,86]. However, LACV NSs did not influence two other markers of the DNA damage response, p53 and BRCA1 [72]. When DNA is damaged, transcriptional arrest of RNA polymerase II occurs and triggers the degradation of RPB1 by the ubiquitin-proteasome system [87–91]. By removing RPB1 from the transcribing RNA polymerase complex, LACV NSs is able to disassemble the whole transcribing complex [72]. Therefore, since LACV activates the RIG-I pathway, LACV NSs counteracts the subsequent IFN gene transcription by shutting down RNA polymerase II dependent transcription and inhibiting the production of IFNs [72]. BUNV, which is closely related to LACV, is able to inhibit transcription in a similar manner through a different cellular target (Figure 3; Table 1).

As with LACV, mutant BUNV lacking the NSs protein is impaired in its ability to shut off host cell protein synthesis in mammalian cells when compared to WT BUNV and is a strong inducer of IFN-β via the dsRNA-activated transcription factors NF-κB and IRF-3 [84,85]. The BUNV NSs inhibits transcriptional activation of the IFN-β gene without affecting the activation of IRF-3 indicating that the NSs inhibitory effect is downstream of the primary signaling pathway [92]. Using another recombinant BUNV lacking the interaction domain of NSs with MED8, Leonard *et al.* found these viruses to have an impaired ability to inhibit host cell protein expression and an inability to inhibit the IFN response [84]. MED8 is an essential component of the Mediator transcription complex, which is involved in RNA polymerase II regulation by recruiting it to the promoter-bound preinitiation complex and interacting with the c-terminal domain (CTD)-serine-2 kinase P-TEFb [93–96]. Both serine 2 and serine 5 of the CTD of RNA polymerase II are the main regulators of polymerase activity. The phosphorylation of serine 2 is critical for mRNA elongation and 3′-end processing. BUNV NSs prevents this phosphorylation suggesting a block in transition from initiation to elongation [84,85,97,98]. Therefore, BUNV NSs inhibits transcription mediated by RNA polymerase II by preventing the phosphorylation of serine 2 of RPB1 (Figure 3; Table 1). LACV NSs specifically targets CTD-serine-2-phosphorylated RPB1 for degradation and therefore requires phosphorylation of serine 2. Thus, orthobunyaviruses counteract the IFN response by preventing the expression of IFNs using their NSs proteins to specifically shut down RNA polymerase II dependent transcription ensuring the antiviral response genes are selectively shutoff. Further experiments are needed to determine if the orthobunyavirus NSs blocks IFN induction solely by host cell transcriptional inhibition or whether NSs has a more specific anti-IFN mechanism.

### 4.2. Rift Valley Fever Virus (RVFV)

Similarly, RVFV NSs inhibits host cell transcription [83], but through a distinct mechanism that results in NSs forming filamentous structures in the nucleus of infected cells [99]. Here, the block of cellular RNA synthesis is due to RVFV NSs targeting TFIIH-dependent transcription. Yeast two-hybrid screening identified the p44 subunit of the TFIIH transcription factor as a target or binding partner of RVFV NSs and further experiments demonstrated that RVFV NSs interacts with p44 in a complex that also contains the XPB subunit of TFIIH [83]. Furthermore, both p44 and XPB subunits of TFIIH, along with RVFV NSs, are main components of the nuclear filamentous structures. However, RVFV NSs does not inhibit the activity of TFIIH nor does it dissociate the TFIIH complex [83]. Le May *et al.* favor the hypothesis that RVFV NSs causes a dramatic decrease in cellular TFIIH concentration. Upon RVFV infection, NSs is translated in the cytoplasm and binds to p44 to be transported to the nucleus. Even though the XPD/p44 interaction is stronger than the NSs/p44 interaction, NSs likely monopolizes this interaction due to its presence in large excess compared to p44 and is likely the reason for the accumulation of XPD in the cytoplasm [83]. In the nucleus, NSs might prevent the assembly of newly synthesized TFIIH and/or recycled TFIIH subcomplexes (Figure 3; Table 1), because NSs is unable to target p44 when present in the core TFIIH or once p44 is interacting with XPD [83]. Two important results begin to elucidate the mechanism of RVFV NSs transcriptional inhibition. First, the time course of infection shows a concomitant decrease in TFIIH subunits p51, p62, and XPD with the synthesis of NSs and the formation of nuclear filament containing p44 and XPB. Second, the naturally avirulent Clone C13 of RVFV, which contains a deleted NSsΔ16-198, replicates and produces viral proteins but does not lead to a decrease in TFIIH concentration or RNA synthesis, suggesting that other viral proteins do not affect the basal transcription machinery [83].

The general inhibition of host cell transcription by RVFV is very potent and begins to occur approximately 8 hours after infection [83]. However, the IFN-β gene is constitutively repressed in cells and is turned on as soon as a virus infects the cell. RVFV is able to inhibit IFN-β gene expression as early as 4 hours after infection [100]. The NSs of RVFV directly interacts with Sin3A associated protein 30 (SAP30), a component of Sin3A corepressor complexes and a partner of the transcription factor YY1 [100]. In uninfected cells, the SAP30 corepressor complex interacts with the constitutively repressed IFN-β promoter at the −90 position through its YY1 binding site. After viral infection, binding of YY1 to its −122 site recruits CBP to the promoter region of the IFN-β gene resulting in IFN-β gene expression [100]. SAP30 colocalizes with RVFV NSs filament as early as 5 hours after infection and RVFV NSs interacts with YY1 of the IFN-β gene promoter as soon as 4 hours following infection. RVFV NSs inhibits IFN-β gene expression by reinforcing the corepressor complex and preventing the recruitment of CBP to the IFN-β promoter (Table 1) [100]. Importantly, these studies demonstrate that YY1 and corepressors can influence many promoter regions. Therefore, RVFV NSs inhibits IFN-β early after infection by interacting with SAP30 then blocks IFN production later during infection by a generalized host cell transcriptional shutoff [83,100]. The early block of IFN-β may have evolved to inhibit early IFN-β that may occur before RVFV NSs is able to interfere with TFIIH since NSs is unable to interfere with already formed TFIIH complexes [83]. Furthermore, the authors speculate that genes whose promoters interact with SAP30 and/or YY1 could be sensitive to abnormal transcriptional regulation by RVFV NSs filaments and could explain some of the pathogenic effects due to RVFV, such as abortion.

IFN antagonists are common among the bunyaviruses and all bunyaviral NSs proteins thus far (RVFV, LACV, BUNV, and sandfly fever Sicilian virus) have been shown to block transcriptional induction of IFN genes and cause cellular protein synthesis inhibition, but are unable to alter the level of double-stranded RNA-dependent protein kinase (PKR) [71,78,85,101–103]. However, RVFV is unique among the bunyavirus NSs proteins because it has the additional ability to degrade PKR, most likely by the proteasome (Figure 1; Table 1) [78]. Poliovirus is the only other example of a virus that has been shown to degrade PKR in a similar manner [104]. While RVFV NSs is the bunyaviruses non-structural protein capable of degrading PKR, members of both the hantaviruses and nairoviruses cleave the triphosphate group off their 5′ genomic end [66]. This 5′ triphosphate group is important since ssRNAs containing a short stem-loop structure have been shown to activate PKR [105] and some of these viruses remove this 5′ triphosphate group to avoid IFN induction via RIG-I [66].

Bunyavirus NSs act as IFN antagonists by quickly and efficiently shutting down host cell transcription by targeting RNA polymerase II or its preinitiation complex (Figure 3; Table 1), as is the case for RVFV NSs [72,83,84]. LACV NSs degrades transcriptionally active RNA polymerase II while BUNV likely prevents the transition of mRNA transcription from initiation to elongation while leading to the degradation of RNA polymerase II [72,84,85,106]. RVFV has similarities to both LACV and BUNV in that it degrades PKR and targets TFIIH, which is a component of the RNA polymerase II preinitiation complex. However, RVFV NSs also leads to a decrease in RNA polymerase I activity as well [83]. Although BUNV NSs has not been examined for inhibition of RNA polymerase I activities, this possibility is unlikely because of its close relation with LACV NSs and the specificity of LACV NSs for RNA polymerase II. The ability of RVFV to inhibit the IFN response by multiple mechanisms and prevent IFN-β induction soon after infection may provide several insights into bunyavirus virulence and pathogenesis. The multifaceted inhibition of IFN induction by RVFV may reflect its more severe disease relative to LACV and BUNV. For example, RVFV NSs has the ability to block the virus from being sensed by PKR, block early IFN-β production, and then lead to general host cell transcriptional shutoff providing ideal cellular conditions for RVFV replication to flourish and subsequently infect surrounding cells since IFN production has been blocked and the neighboring cells have not achieved an antiviral state. Moreover, while it has not been demonstrated experimentally, RVFV NSs may target early IFN-β expression to counteract its detection by PKR, TLR3 or another viral sensor that signals through TRIF to quickly induce IFN-β.

## 5. Alphaviruses

The *Alphavirus* genus of the *Togaviridae* is composed of about 30 members that are grouped by geographic distribution into Old World and New World alphaviruses [107]. Similar to the *Bunyaviridae*, the alphaviruses can infect a variety of invertebrate and vertebrate hosts, including humans, with most being transmitted by mosquitoes [108]. Infection of invertebrate vectors results in persistent, lifelong infection and viral replication without adversely affecting the host. However, infection of susceptible vertebrate cells leads to rapid cytopathic effects and vertebrate hosts usually develop acute infection resulting in disease. Alphaviruses cause a wide range of disease with most Old World alphaviruses causing arthritis and rash illness and New World alphaviruses leading to encephalitis. However, the distinction of Old and New World alphaviruses based on disease outcomes is not clear and some Old World alphaviruses have been associated with neurologic disease; Sindbis virus (the prototype alphavirus) causes encephalomyelitis in mice and Ross River virus (RRV) and chikungunya virus (CHIKV) cause neurological disease in humans and equines [109].

Alphaviruses are positive-strand RNA viruses with genomes of approximately 12 kb, encoding two polyproteins. The polypeptide encoded at the 5′ end of genome is composed of the nonstructural proteins (nsPs) 1, 2, 3, and 4 involved in viral replication as the nsP123 or nsP1234 precursor that function in a complex for viral negative-strand RNA synthesis. nsP123 is processed into its individual proteins and the nsPs function to replicate the viral genome [108]. The mature nonstructural proteins, together with nsP4 form the replication complex. Here, nsP1 serves as the methyltransferase and guanylyltransferase while nsP2 has a papain-like protease domain and helicase activity. The RNA-dependent RNA polymerase is encoded as nsP4 [108]. Encoded downstream of the replicase is the second polyprotein, which contains the structural proteins: the capsid protein and envelope glycoproteins [108]. As with many viruses in mammalian cells, alphavirus replication usually results in translational shutoff of the host cell leading to the suppression of innate immunity [110]. However, this host protein synthesis shutoff alone is not sufficient for alphaviruses to achieve a productive infection [111]. New World and Old World alphaviruses have been demonstrated to use different mechanisms to achieve host cell translational shutoff, with Old World alphaviruses using nsP2 and New World alphaviruses utilizing the capsid protein. However, recent evidence suggests New World alphaviruses may have additional methods to counteract the host IFN response.

### 5.1. New World Alphaviruses

EEEV and VEEV (New World alphaviruses) shut off host cell transcription, which involves the capsid protein, through mechanisms that are still unclear. There is evidence that the capsid protein’s activity is associated with defects in type I IFN and ISG induction, most likely due to global transcriptional shutoff [16,17,112]. However, recent experiments using VEEV replicons demonstrated that VEEV disrupts the cellular response to IFNs by mechanisms that do not require the viral capsid [111]. Since these replicons do not contain any structural genes and cells infected with these replicons do not activate STAT1, it is likely that the expression of a viral nonstructural protein is responsible for this shutoff. The inhibition of STAT1 activation prevents the nuclear localization of STAT1 and subsequently results in decreased STAT1-dependent gene transcription [111]. Global host cell transcriptional and translational shutoffs probably work synergistically with STAT1 inhibition; it is likely that host shut off is not required for the inhibition of JAK/STAT signaling by VEEV replicon expression. The STAT1 antagonizing mechanism decreases STAT1 phosphorylation (Figure 1; Table 1) but does not affect the overall STAT1 levels and the IFNAR complex is still able to activate Jak1, Tyk2 and STAT2, indicating that the mechanism is specific for STAT1 [111]. The mechanism(s) of the block in STAT1 phosphorylation by VEEV has not been elucidated, but Simmons *et al.* [111] suggest that a viral nonstructural protein could bind STAT1 or the receptor complex preventing STAT1’s phosphorylation by JAK kinases and/or its association with the IFNAR.

### 5.2. Old World Alphaviruses

Recently, it has been shown that JAK-STAT signaling is inhibited by the CHIKV nsP2 overexpression. Notably this is the first time that an alphavirus nsP2 alone has been shown to be sufficient for this inhibition [113]. Previously, it was known that IFNAR-dependent type I IFN signaling was required to limit CHIKV replication in animals and IFN-α inhibited CHIKV replication in mice when administered prior to, but not after, CHIKV infection [114]. As with VEEV, IFN antagonism by CHIKV is independent of host cell shutoff since cells treated with cycloheximide, an inhibitor of cellular protein synthesis, did not have altered JAK-STAT signaling nor reduced endogenous levels of STAT1 [111,113]. Additionally, the authors suggest that nsP2 is such a potent inhibitor of STAT1 nuclear translocation (Figure 1; Table 1), any other contributory activity by other viral proteins may not be needed to establish a productive infection and STAT1 up-regulation in CHIKV-infected cells is prevented by the inhibition of JAK-STAT signaling [113]. Recently, CHIKV was shown to strongly induce the transcription of IFN and ISGs via IPS-1; however, these mRNAs are not translated into protein, as there is a widespread block of cellular, but not viral, translation [115]. Although the viral factor(s) responsible for this translation block are currently unknown, it may represent a novel mechanism of translational block since it occurs independently of PKR [115]. Furthermore, a late shutoff of host cell RNA synthesis induced by CHIKV prevents the transcription of IFN-β and ISGs [115].

A mutation of a conserved proline in CHIKV nsP2 at position 718 prevented a block of IFN-β-induced STAT1 nuclear translocation. Similar results were obtained for SINV replicons with a mutation at a conserved proline at position 726 suggesting these viruses use similar mechanisms to block the JAK-STAT pathway [113]. These experiments were based on the previously characterized mutation of P726 in SINV that resulted in noncytopathic RNA replication [116] and attenuated virus growth associated with high IFN production [116,117]. More recent studies confirmed that infection with WT SINV led to minimal up-regulation of transcription from genes driven by the IFN-β promoter. However, IRF3 still dimerized, translocated to the nucleus and bound DNA [118]. In contrast, a noncytopathic SINV (39nc) did not block the release of IFN-α/β. This IFN production was dependent on the presence of TBK1 and was likely associated with activation of the NF-κB-dependent PRDII promoters (Table 1) [118]. Additionally, MDA5 is partially responsible for IFN induction by SINV 39nc and is likely activated by the high levels of dsRNA produced during alphavirus replication [118,119].

A mutant Semliki forest virus (SFV) with a single point mutation in the RRR (wt) nuclear localization within nsP2 has provided more evidence for the alphavirus nsP2 as a potent and specific inhibitor of the IFN response. This mutant SFV replicates comparably to wt SFV and induces similar levels of global transcription and translational shutoff in the cell. However, it induces significantly more type I IFN, indicating that the differences in cytokine responses are not solely due to the global suppression of the host cell [120]. Although the precise mechanism(s) of this IFN antagonism have not been determined, the nuclear translocation of IFN-inducing transcription factors IRF-3 and NF-κB is unaffected, suggesting that SFV nsP2 suppresses type I IFN induction at some point beyond the nuclear translocation of these transcription factors [120]. However, TNF-α levels were also increased in mutant SFV infected cells indicating a non-IFN-specific effect on host cell gene expression. To explain this phenomenon, Breakwell *et al.* suggest that nsP2 could inhibit larger groups of genes by blocking the activity of a particular transcription factor [120]. One possible explanation of the inhibitory effects of nsP2 on the host cell responses could stem from nsP2′s papain-like protease domain. In the replication of SFV, this domain of nsP2 processes the nsp1234 polyprotein and leads to the viral replicase complex [121,122]. Since nsP2 translocates to the nucleus, this protease activity could be maintained and result in the cleavage of cellular transcription factors (Table 1) similar to that seen in the rhinovirus 3C protease [123]. Further experiments using constructs of SFV nsP2 with deletion or mutation of the papain-like protease domain could be used to explore this possible mechanism of inhibitory action.

More recent studies of SINV and RRV with mutations in the nsP1/nsP2 cleavage domains of nsP1 have demonstrated a role for nsP1 in modulating type I IFN induction independently of host cell shutoff [124]. In contrast to the studies of SFV in which IRF-3 were unaffected, these SINV and RRV mutants affected IRF-3 (Table 1) suggesting different mechanisms of IFN antagonism [124]. Even though these inhibitory mechanisms have not been defined, these observations support a model in which alphavirus nsPs play a major role in modulating type I IFN induction independent of host cell shutoff, and likely by using multiple and distinct mechanisms.

## 6. Flaviviruses

Flaviviruses encode seven nonstructural proteins (NS1, NS2A, NS2B, NS3, NS4A, NS4B, and NS5) with several of these nonstructural proteins involved in the flaviviral replication complexes [125–128]. Some of these flaviviral nonstructural proteins are involved in counteracting the IFN response and may have evolved separately as the flaviviral lineages diverged early in the evolution of the genus. Of the 75+ viruses within the *Flavivirus* genus, approximately 40 are mosquito-borne, 16 are tick-borne, and 18 have no known vector [129]. Furthermore, the three evolutionary lineages can be separated by genomic sequence analysis and base substitution rates [130]. While NS5 is the main virulence factor for tick-borne flaviviruses [131,132], mosquito-borne flaviviruses use NS5 as well as other non-structural proteins to inhibit the IFN response (NS2A, NS2B, NS3, NS4A, and NS4B) [133–136].

### 6.1. Tick-Borne Flaviviruses

The tick-borne encephalitis serocomplex of flaviviruses includes tick-borne encephalitis virus (TBEV), Powassan, and Kyasanur Forest disease virus. Members of this serogroup are an emerging arboviral threat due to the high morbidity and mortality rates associated with disease, including encephalitis, meningitis, and/or hemorrhagic fever. TBEV is transmitted by Ixodes ticks and, more rarely, in infected milk (goat, sheep, and cow milk) and aerosols [137]. TBEV is endemic in Russia, Czech Republic, Austria, Poland, Serbia and Montenegro [138]. However, in recent years, TBEV has spread to Italy, Denmark, Scandinavia, and Greece demonstrating the emergence potential of TBEV and other related tick-borne flaviviruses [138–140].

Langat virus (LGTV), a member of the TBE serocomplex, is sensitive to the antiviral effects of IFN when added exogenously prior to infection in VERO cells [132], which can respond to but not produce IFNs [141]. However, when LGTV infection occurred prior to IFN addition, phosphorylation of STAT1, STAT2, Tyk2, and Jak1 were all inhibited [132], suggesting that LGTV must establish a productive infection to counteract the IFN response. In these LGTV infected cells, levels of STAT1 and STAT2 were unchanged but the phosphorylation of STAT1 in response to IFN-α or IFN-γ stimulation, as well as STAT2 in response to IFN-α, were inhibited [132]. Expression of individual LGTV nonstructural proteins identified NS5 as the direct IFN antagonist and demonstrated its ability to suppress IFN-stimulated STAT1 phosphorylation by itself (Figure 1; Table 1). This inhibition of JAK-STAT signaling is likely due to the direct association of NS5 with IFNAR2 and/or IFNGR1, allowing for early virus replication in individual cells [132]. The direct association of NS5 with IFNAR2 subunits suggests a host factor for proper targeting of NS5 to the plasma membrane where the receptors are located.

A yeast two-hybrid screen using TBEV NS5 against a human brain cDNA library identified scribble (hScrib) as a binding partner [131]. hScrib is in the LAP (leucine-rich repeats and PSD/Dlg/ZO-1 [PDZ]) family of proteins [142] and plays a role in T-cell polarity, which is required for T-cell activation and antigen presentation [142]. Furthermore, hScrib is thought to stabilize cell-to-cell contacts involving E-cadherin at the membrane of polarized mammalian cells, supporting it as a potential cellular chauffeur of NS5 to the cell membrane [143]. TBEV NS5 colocalizes with hScrib and E-cadherin at cell-to-cell contacts in mammalian cells. Importantly, hScrib knockdown in NS5 expressing cells rescued phosphorylation and nuclear translocation of STAT1 [131]. When direct binding of the methyltransferase domain of TBEV with the fourth PDZ domain of hScrib was disrupted by a binding-defective mutant NS5, the nuclear phosphorylated STAT1 was partially restored and NS5 was unable to accumulate at the cell surface [131].

Among the TBE virus serogroup, NS5 has 80–90% amino acid identity, suggesting the conservation of the IFN antagonist mechanism by NS5 within the serogroup. However, the interaction of LGTV NS5 with hScrib is still unresolved. Although it has been suggested that the mechanism of IFN antagonism by NS5 of the serogroup is conserved among the flaviviruses, experimental and amino acid identity suggest a similar yet different mechanism(s) among the mosquito-borne flaviviruses. The NS5 highly conserved amongst members of the TBE serogroup and shares 60% amino acid identity with the NS5 of WNV and DENV-2. The divergence of tick-borne and mosquito-borne flaviviruses NS5 reflects the phylogenetic divergence of these groups and their strategies to counteract host IFN.

### 6.2. Mosquito-Borne Flaviviruses

The mosquito-borne flaviviruses are arguably the best-known arboviruses and include WNV, YFV, and DENV. The most widespread of arboviruses, DENV is responsible for 50 million to 100 million infections annually with almost half of the world’s population living in an at-risk area. There are four DENV serotypes (DENV1-4) and infection typically results in a flu-like illness with life-long immunity to that serotype. However, infection with another serotype can lead to the more severe dengue hemorrhagic fever or dengue shock syndrome. The increase in vascular permeability is perhaps the most serious complication of dengue hemorrhagic fever or dengue shock syndrome, and it has been suggested that infected immature dendritic cells overproducing matrix metalloproteinase (MMP)-9 are responsible for this permeability [144]. Thus, the cross-reactivity among DENV serotypes and the possible role of MMP-9 in severe DENV disease underscores the importance of understanding these aspects of host virus interactions as they will guide the development of therapeutics and vaccines.

Cytoplasmic DENV NS5 expressed alone is sufficient to efficiently inhibit the IFN-α, but not IFN-γ response. More specifically, cells expressing DENV NS5 alone inhibited STAT2, but not STAT1, phosphorylation without affecting levels or phosphorylation of Tyk2 or surface expression of IFNAR2 [134]. Since STAT2, but not STAT1 or Tyk2, coimmunoprecipitates with NS5, the phosphorylation and activation of STAT2 is prevented by either direct or indirect association of NS5 and preventing STAT2′s interaction with Tyk2 [134]. Interestingly, when cells are infected by DENV or replicons expressing all the nsPs, STAT2 levels are decreased and this decrease requires intact proteasome and ubiquitinating activities [133,134]. A protease cleavage signal upstream of the N-terminus of DENV NS5 is required for STAT2 to be targeted for degradation. However, the protease required for maturation of NS5 with concomitant degradation of STAT2 does not seem to be specific (Figure 1; Table 1). Therefore, the NS5 precursor being cleaved to mature NS5 is required for the degradation of STAT2 [133,134]. The mechanism of STAT2 degradation is still unclear beyond the involvement of the proteasome and ubiquitination [133,134]. Since DENV NS5 prevents IFN signaling, the degradation of STAT2 may provide an additional advantage to the virus.

While DENV, LGTV, and TBEV block JAK-STAT signaling by direct binding to one of the main signaling components, JEV NS5 targets protein tyrosine phosphatases (PTPs) to exert their IFN antagonist effect [145]. PTPs are one of several cellular negative regulators of the JAK-STAT signaling pathway along with protein inhibitors of activated Stats (PIAS) and suppressor of cytokine signaling (SOCS) proteins. JEV NS5 effectively blocks STAT1 and Tyk2 phosphorylation (Figure 1; Table 1) and sodium orthovanadate, a broad spectrum inhibitor of PTPs, abolishes the block in STAT1 and Tyk2 phosphorylation [145]. The use of specific PTP inhibitors did not block the effect of JEV NS5, suggesting the responsible PTP is probably an unidentified cellular PTP [145]. JEV NS5 itself could act as a PTP but sequence alignment with PTPs suggests that JEV NS5 does not possess PTP activity. Furthermore, neither the methyltransferase or polymerase activity of JEV NS5 alone appears to be responsible for counteracting JAK-STAT signaling [145]. Therefore, JEV NS5 does not block the induction of IFN, but activates a cellular PTP to dephosphorylate STAT1 and Tyk2, thus preventing the nuclear translocation of STAT1.

DENV NS5 is not the only nonstructural protein encoded by DENV that has IFN antagonizing activities. Individual expression of DENV NS2A, NS4A, and NS4B enhanced replication of IFN-α/β sensitive Newcastle disease virus [135]. Furthermore, each of the three nonstructural proteins inhibited activation of two different ISRE promoters, albeit NS2A and NS4A caused less inhibition than NS4B. When all three of these nonstructural proteins were coexpressed, IFN signaling was almost completely abrogated suggesting a synergistic and cooperative IFN counteraction by these viral proteins [135]. As with the ability of DENV NS5 to degrade STAT2, NS4A/NS4B cleavage is required for the anti-IFN activity of NS4B. Additionally, NS4B’s localization and insertion into the ER membrane are required for NS4B anti-IFN activity since the absence of a signal peptide renders NS4B nonfunctional (Table 1). The N-terminal 2K segment of DENV NS4B is not involved in the anti-IFN activity, as it can be replaced by another signal peptide without altering NS4B functionality [135].

The precise mechanism of DENV NS4B has not been determined, although it does prevent the nuclear translocation of STAT1 in response to IFN-β [135]. Interestingly, Muñoz-Jordán *et al.* determined the amino acids in region 77 to 125 are required for DENV NS4B to antagonize IFN and within this region, amino acids 77 to 103 are predicted to be cytoplasmic anchored by the first and second transmembrane domains of NS4B [135]. Thus, it would be plausible for these amino acids to interact with cellular components in the cytoplasm involved in IFN-signaling. However, further experiments will be needed to elucidate the interactions of this cytoplasmic domain. Other mosquito-borne flaviviruses, namely YFV and WNV, have conserved NS4B to inhibit the JAK-STAT pathway, suggesting a converging mechanism of the NS4B proteins among the mosquito-borne flaviviruses.

Because many viruses become sensitive to the IFN response when their IFN antagonist’s function is disabled, these nonstructural proteins are promising targets for the development of attenuated viral vaccines. The introduction of the NY 99 strain of WNV (WNV_NY99_) has gained considerable attention due to its recent emergence and rapid spread in the United States. While WNV_NY99_ is highly virulent, its close relative, Kunjin virus (WNV_KUN_), exhibits a highly reduced pathogenesis in humans, animals, and birds. Even though these two viruses differ greatly in their virulence, they are more than 98% identical in their amino acid sequences and express all seven flavivirus nonstructural proteins, making WNV_KUN_ a possible candidate for the development of a WNV vaccine [146]. Therefore, the differences in virulence are likely due to mutations in WNV virulence factors. Furthermore, immunization with WNV_KUN_ completely protects mice from a lethal challenge with WNV_NY99_ [147]. However, further attenuation of WNV would be ideal for a safe, live WNV vaccine. One example of further attenuation in the context of WNV vaccine candidates is a mutation in NS2A, which is a major suppressor of IFN-β transcription. A single amino acid mutation in NS2A (alanine to proline at position 30; A30P) prevented this suppression of IFN-β and induced IFN-β transcription ∼6–7-fold more efficiently than WT WNV_KUN_ [136]. Although WNV_KUN_NS2/A30P did not grow or spread as well as WT WNV_KUN_ in IFN-α/β producing cell lines, the levels of IFN-α/β were higher in the cell culture supernatant of those cells infected with the mutant virus [148]. When mice were immunized with WNV_KUN_NS2/A30P, a strong humoral response was induced, indicating that this mutation did not compromise the magnitude of immune response mounted against the virus. Furthermore, the immunization provided complete protection to all of the mice against peripheral challenge of a lethal dose of WNVNY99 [148]. The mutant virus was also attenuated after intracranial inoculation, which is not surprising since encephalitic viral infections induce IFN responses in the brain. While the LD_50_ of WNV_KUN_NS2/A30P was almost 100,000 fold higher than WNV_KUN_ after peripheral inoculation, the difference in LD_50_s of the two viruses was less profound following intracranial inoculation [148]. These studies provide promising evidence for the further attenuation of vaccine candidate viruses by targeting their IFN antagonist nonstructural proteins, but also demonstrate the need to better understand the interactions of arboviruses and the host immune system, particularly in the CNS.

## 7. Conclusions

The mammalian IFN response is an important antiviral defense mounting both innate and adaptive immune responses to control and clear viral infections. However, better models of these host-virus interactions need to be developed. As previously mentioned, most inbred strains of mice have defective Mx2, the homologue of human MxA, which is the most effective antiviral effector protein described in humans against LACV. Further work is needed to evaluate the efficacy of interferon responses in mouse models. Moreover, parallel experiments *in vitro* in human cells may help in determining the reliability or transferability from mouse models to humans of such findings. While many large-scale factors, e.g., climate change, population growth, travel, and urbanization, have contributed to the emergence and re-emergence of many arboviruses, perhaps one of the most constant features of emerging arboviruses is their widespread use of virus-encoded nonstructural proteins to counteract the IFN response. As more viral IFN antagonists are being identified and characterized, it is clear that many viruses use a variety of mechanisms to counteract the host-immune response. Furthermore, these studies have provided more experimental evidence to support the hypothesis that severity of disease associated with many arboviral infections correlates with the ability of the virus to counteract the IFN response. The ability of some viruses, like RVFV, to counteract multiple pathways (Table 1) in its detection or in the induction or signaling of IFNs likely enhances the pathogenicity of the virus. A better understanding of host-virus interactions and the viral determinants responsible for disease outcome and virus emergence and re-emergence are a prerequisite for the development of attenuated viral vaccines and anti-viral therapies that will both control the spread of these emerging infections and lead to better prophylaxis and health outcomes.

## Figures and Tables

**Table 1.** Direct and indirect interferon antagonist functions of arboviral nonstructural proteins.

**Family** ** Genus** ** Virus**	**Nonstructural Proteins**	**Direct IFN Antagonism**	**Host Transcriptional Shut off as Indirect Mechanism for IFN Antagonism**	**References**
*Bunyaviridae*				
*Orthobunyavirus*				
La Crosse virus	**NSs**		-Inhibits RNA Pol II transcription by triggering degradation of RPB1	[72]
Bunyamwera virus	**NSs**		-Inhibits RNA Pol II transcription by blocking elongation	[84,85]
*Phlebovirus*				
Rift Valley Fever virus	**NSs**	-Degrades PKR -Interacts with SAP30 to block activation of the IFN-β promoter	-Inhibits RNA Pol II transcription by preventing TFIIH assembly	[78,83,100]
*Togaviridae*				
*Alphavirus*				
Venezuelan Equine Encephalitis virus	**nsP2?**	-Blocks STAT1 phosphorylation	-Transcriptional shutoff by capsid protein	[17,111]
Chikungunya virus	**nsP2**	-Blocks STAT1 nuclear import	-Transcriptional shutoff (mechanism unknown)	[113,115]
Sindbis virus	**nsP2**	-Blocks NF-κB-dependent PRDII promoters?	-Downregulation of RNA Pol I and II-dependent transcription	[16,118]
Semliki Forrest virus	**nsP2**	-Cleavage of transcription factors (suggested)	-Transcriptional shutoff (mechanism unknown)	[16,120]
Ross River virus	**nsP1**	-Block IRF-3?		[124]
*Flaviviridae*				
*Flavivirus*				
Langat virus	**NS5**	-Blocks STAT1 phosphorylation by interaction with IFNAR2/IFNGR1		[132]
Tick-borne Encephalitis virus	**NS5**	-Blocks STAT1 phosphorylation by a mechanism involving interactions with hScrib		[131]
Dengue virus	**NS5**	-Blocks STAT2 phosphorylation -Degrades STAT2		[133,134]
	**NS4B**	-Unknown but requires localization and insertion into ER membrane		[135]
	**NS2A/NS4A**	-Unknown		[135]
Japanese Encephalitis virus	**NS5**	-Blocks STAT1 and Tyk2 phosphorylation by activating PTP(s)		[145]
